# Source-related smart suspect screening in the aqueous environment: search for tire-derived persistent and mobile trace organic contaminants in surface waters

**DOI:** 10.1007/s00216-020-02653-1

**Published:** 2020-05-08

**Authors:** Bettina Seiwert, Philipp Klöckner, Stephan Wagner, Thorsten Reemtsma

**Affiliations:** 1 Department of Analytical Chemistry, Helmholtz Centre for Environmental Research – UFZ, Permoserstrasse 15, 04318 Leipzig, Germany; 2 Institute for Analytical Chemistry, University of Leipzig, Linnéstrasse 3, 04103 Leipzig, Germany

**Keywords:** Traffic, PMT, Rivers, Vulcanization accelerator, Leachables, Rainfall

## Abstract

A variant of suspect screening by liquid chromatography–high-resolution mass spectrometry (LC-HRMS) is proposed in this study: Samples of a potential source of contamination and of an environmental sample close to this source are first analyzed in a non-targeted manner to select source-related suspects and to identify them. The suspect list compiled from such an exercise is then applied to LC-HRMS data of environmental samples to ascribe and to identify persistent and mobile contaminants in the water cycle that may originate from the source under study. This approach was applied to tire crumb rubber (source) and road dust (close to source); by comparison of the two data sets, 88% of the features detected in tire leachate could be excluded. Of the 48 suspects remaining, a total of 41 could be tentatively identified as either related to hexamethoxymethyl melamine or cyclic amines, benzothiazoles, or glycols. Subsequently, environmental samples were searched for these suspects: 85% were determined in an urban creek after a combined sewer overflow and 67% in the influent of a municipal wastewater treatment plant (WWTP). These exceptionally high rates of positive findings prove that this source-related smart suspect screening effectively directs the effort of selecting and identifying unknown contaminants to those related to the source of interest. The WWTP effluent and the urban creek during dry weather also showed the presence of numerous contaminants that may stem from tire and road wear particles (TRWP) in road runoff. Contribution from other sources, however, cannot be ruled out.

**Electronic supplementary material:**

The online version of this article (10.1007/s00216-020-02653-1) contains supplementary material, which is available to authorized users.

**Figure:**
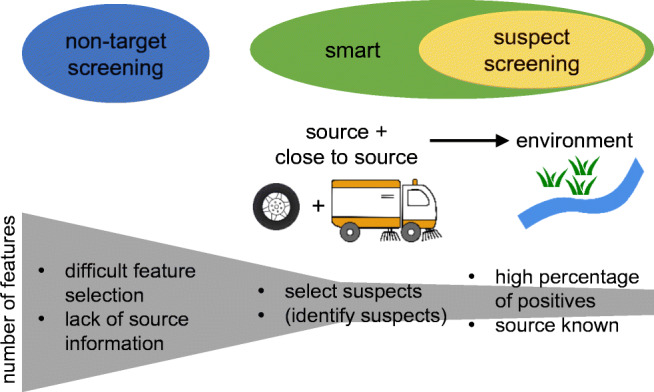
Graphical abstract

## Introduction

Screening for previously unrecognized contaminants in (surface) waters may be done by non-target screening or suspect screening involving liquid chromatography–high-resolution mass spectrometry (LC-HRMS). While non-target screening makes use of the entity of mass spectrometric data collected over a chromatographic run, its data processing effort is extremely high and its success often limited by the need to effectively reduce the data set and by the difficulty to identify unknown compounds based solely on their mass spectrometric data.

In comparison, suspect screening is more straightforward as it makes use of existing (chemical) knowledge, such as on compounds that may be expected to be of relevance for the environmental compartment being studied, e.g., because they have already been detected in such settings previously. Suspect screening may even account for transformation products (TPs), as long as those are formed along known transformation pathways. Correspondingly, it was concluded recently in a study on pesticide metabolites in groundwater that suspect screening was more successful than non-target screening [1].

In Europe, most chemicals sold by industry, whether to the consumer or for use in further production, have to be registered under REACH. Parts of the information provided by industry in the registration process are publicly available; it is a rich source of information also for suspect screening [2]. For example, REACH data has recently been used to select and prioritize potential persistent and mobile (PM) substances of environmental relevance [3] and, combined with tonnage and use information, resulted in a list of more than thousand PM compounds to search for [4].

However, chemicals may be altered in further production processes or may age during use of a product, so that not the parent chemical but its TPs are released into the environment. Such TPs are usually unknown and cannot be included in suspect screening.

Laboratory experiments are useful to study biotic and abiotic transformation processes and allow to decipher TPs by LC-HRMS analysis [5]. One approach involves the use of non-target screening of laboratory experiments and of environmental samples and to create a list of common signals that originate from the transformation process under study—an approach that was called “smart suspect screening” [6]. Smart suspect screening differs from “conventional” suspect screening in the way that the suspects are not compiled from literature or predicted by a software but generated by a combined experimental approach; it works even for non-identified TPs. For example, this approach has been used to cover putative TPs of carbamazepine and lamotrigine in suspect screening by (i) performing different abiotic transformation experiments, (ii) compiling the detected TPs in a suspect list, and then (iii) searching for the occurrence of these TPs in environmental samples [6]. In doing so, smart suspect screening stands between non-target and “conventional” suspect screening.

After having detected a novel contaminant in aqueous environments, knowledge of its source is, obviously, a prerequisite to reduce its release. While information on the potential use of chemicals may be found, sometimes in data bases of suspects [7], many chemicals are used in very diverse applications [8] so that use information may not help much to identify relevant sources. In the case of TPs, source information is, obviously, even more difficult if not impossible to obtain. Therefore, non-target screening, even if successful in terms of identifying a contaminant, may not allow to deduce instructional knowledge that would allow one to react on it.

Here, a variant of smart suspect screening is proposed, which aims at generating information on environmentally relevant sources of substances and their TPs. This “source-related smart suspect screening” can either be used to assess a certain emission source with respect to its relevance for the compartment under study (e.g., surface water) or to trace contaminants found in an environmental compartment back to their source, even if the contaminant is an aging or degradation product.

This source-related smart suspect screening is illustrated by its application to automobile tires and tire and road wear particles (TRWP) as an important traffic-related emission into the environment [9–11]. While tires primarily consist of rubber and fillers, they also contain a large diversity of organic chemicals that are required for tire production or for maintaining the tire quality during use and that amount to 5–10% of tire weight [9]. Among them are vulcanization accelerators, activators, plasticizers, processing aids, and antioxidants. These substances are partially transformed during tire production or use. Consequently, not only the parent chemicals but also their aging or degradation products may later be released from tires or TRWP into the aqueous environment [12]. Some of these TPs are known for long, e.g., benzothiazoles [13], while others, such as hexamethoxymethyl melamine (HMMM) have been recognized only recently [14, 15].

However, release of persistent and mobile substances from tires or TRWP into aqueous environment has not been intensively studied, thus far [15]. And while a number of tire ingredients are known, the information on the products formed from them during tire production and use is also limited [12]. Taken together, the information available on organic trace contaminants that may be found in the aquatic environment and that originate from tires is limited. Such knowledge is of relevance, however, because such compounds would spread independently from TRWP in the aqueous environment [13].

Therefore, this study illustrates the concept of source-related suspect screening for tire-related organic contaminants. It explores the use of this screening approach by applying it to urban surface waters, especially waters affected by combined sewer overflow and to municipal wastewater of a combined sewer system that receives road runoff.

## Materials and methods

### Laboratory leaching experiments

A sample of tire crumb rubber (TCR) was obtained from a rubber recycling company (PVP Triptis, Triptis, Germany). The sample consisted of shredded car tires (grain size < 600 μm) of which the textile and steel cords were removed.

A road dust (RD) sample was collected from a street-sweeping car in the City of Leipzig, Germany. The car collected road dust over a total sweeping distance of 38 km. For the swept roads, an average of 226,700 cars per day was determined in 2016. Water spray was used while sweeping for increased collection efficiency. The sample was taken on April 19, 2019, after clearance of the cars’ dust container and before water was drained from the road dust. Afterwards, the sample was frozen, lyophilized, and sieved over a stainless steel mesh (250 μm, Retsch, Haan, Germany).

Leaching of the TCR and RD sample was performed with a citrate-phosphate buffer solution (pH 5.2) in batch using 50-mL centrifugation tubes (Nunc, Thermo Scientific, Waltham, USA) in an overhead shaker (214/12, Guwina-Hoffmann, Berlin, Germany) at a speed of 50–60 rpm over 48 h. The liquid-to-solid ratio was 2 g/L for TCR and 200 g/L for RD.

### Environmental samples

Three samples were collected in the urban river Parthe within the city of Leipzig, Germany (51° 21′ 23.8″ N 12° 20′ 57.8″ E). The sampling took place in autumn/winter period 2016/2017. Sampling dates were chosen according to hydrological conditions covering low, medium, and high river discharge [16]. The samples were stored in glass vials and frozen at − 20 °C.

Three sets of samples of the influent (effluent of the primary clarifier) and effluent (effluent of the final settling basin) of a municipal wastewater treatment plant were collected in March and April 2017 over 24 h as a composite sample, filtered over 0.45-μm membrane filters (regenerated cellulose) and stored at 8 °C in a refrigerator until analysis. The WWTP performed a tertiary treatment for 550,000 population equivalents. The samples were collected by and analyzed with permission of the WWTP operators.

### Analysis

LC-HRMS screening was carried out with an ACQUITY UPLC system that was coupled to a Xevo G2-XS mass spectrometer equipped with an electrospray ionization source (Waters Corp., Milford, USA). Chromatographic separation was performed using an ACQUITY UPLC HSS T3 column (100 × 2.1 mm, 1.8 μm) at a flow rate of 0.45 mL/min and a column temperature of 45 °C. The mobile phase consisted of (A) 0.1% formic acid in water and (B) 0.1% formic acid in methanol. The gradient was as follows: 0 min 2% B, 12.25 min 99% B, 15.00 min 99% B; 15.10 min 2% B, 17.00 min 2% B. The injection volume was 10 μL.

Full-scan spectra were collected from *m*/*z* 50–1200 in positive and negative modes (centroid). Source conditions were set as follows: temperature 140 °C, capillary voltage − 0.8 kV, desolvation temperature 600 °C, sampling cone voltage 20 V, and source offset 80 V. Nitrogen was used as the cone gas and argon as the collision gas. The cone gas flow was 50 L/h and desolvation gas flow 1000 L/h. To ensure accurate mass precision during MS analysis, leucine encephalin was infused via the reference probe as lockspray, and a two-point calibration was applied. Two MS data sets were collected in parallel using low collision energy (6 eV, effectively the accurate mass of parent ions) and high collision energy (15–40 eV, fragment ions) in order to obtain the greatest extent of structural information on each suspect.

### Data processing and identification of suspects

For identification of suspects, a peak picking was performed by MarkerLynx. The following parameters were used: retention time 1 to 12 min, mass range *m*/*z* 50–1200, XIC window 0.05 Da, retention time window 0.05 min. The average of the six blank samples and the three replicates of RD and three replicates TCR samples was calculated and the standard deviation was determined, for each of them. Markers were selected that have a standard deviation of lower than 50% and a relative peak area higher than 0.1 for TCR and 0.05 for RD. The marker list was reduced to markers that appear in the extracts with a peak area that is 200 times higher compared with the blank. The marker list was filtered for signals that appear in both the RD and the TCR extracts. It was checked that peaks have a minimum *S*/*N* > 3 and in-source fragments and adducts were assigned to its molecular ion.

The assignment of the chemical formula of precursor and fragment ions was assisted by MassLynx. The criteria were set as follows: mass tolerance ≤ 5 ppm and element limits of C (0–100), H (0–100), O (0–30), N (0–10), S (0–5), P (0–2), and Cl (0–2). If more than one formula matched the criteria, a plausibility check was performed by inspecting the fragmentation pattern.

## Results and discussion

### General concept

**Fig. 1:**
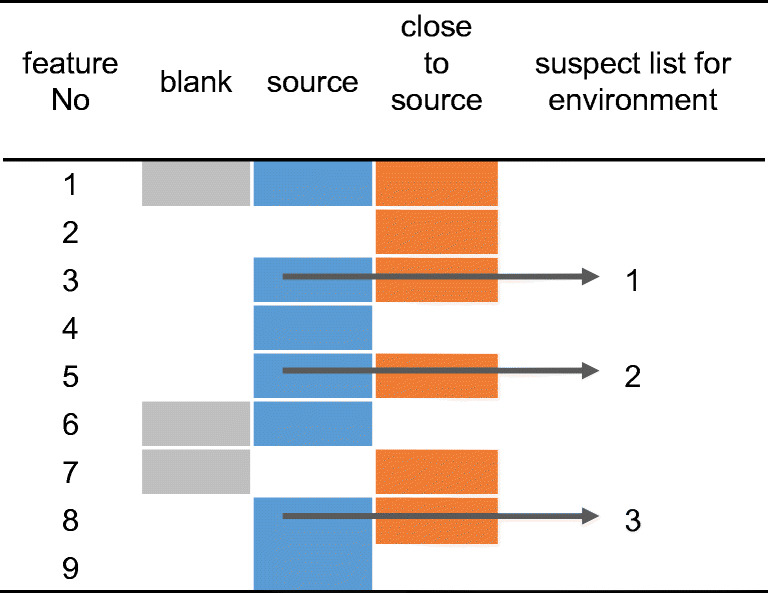
Scheme illustrating how non-target screening of a “source” (blue) and a “close to source” (orange) sample provides a list of suspects for a source-related smart suspect screening in environmental samples. Features detected also in the blank at relevant intensity (gray) are excluded (for details, see text)

To overcome limitations of non-target screening such as (a) the difficult selection of relevant features that merit further attempts of processing and structure elucidation, (b) to provide source-related information, and (c) to link TPs released into the environment to certain sources, a variant of suspect screening is proposed here that can be called source-related smart suspect screening (Fig. 1).

The respective workflow starts with the non-target screening of samples of a potential source and of one or more environmental sample close to this source. The second sample (close to source) acts as a filter for the source data, sorting out signals which are of no relevance for the environment and, hence, not useful in further suspect screening. This may apply to compounds that are of very limited stability or have a higher sorption tendency or are otherwise not transferred into the environment. These two (sets of) samples are processed and analyzed, together with a blank sample. For feature selection, these three samples are considered and only those features are selected for further processing (e.g., identification), which occur in both (sets of) samples but do not occur in the blank (or at significantly lower concentration) (Fig. 1).

The features obtained by this filtering process, whether identified or not, are then compiled in a suspect list and used for suspect screening in environmental samples that may have a larger distance from the respective source but are possibly affected by this as well as other sources (Fig. 1).

This approach is expected to combine advantages of non-target screening and of suspect screening. Compared to usual suspect screening,

- this is a more open approach, considering also previously unrecognized or unknown contaminants, covering also unknown TPs of parent substances formed during production or aging of the product, prior to release into the environment and
- it even allows to detect TPs formed later on in the environment in cases where characteristic fragment ions occur that point at specific structural elements of the parent compounds.

Compared to non-target screening,

- it introduces a rational criterion for selecting features of interest, rather than criteria like signal intensity, which may be unfounded and misleading;
- it reduces the effort in the time-consuming attempts to identify unknowns as only those unknowns will be processed further that were selected in the filtering process and are, thus, relevant for the source of interest;
- it supports the identification process as a higher priority can be given to source-related structure proposals; and
- it provides information on the source of a contamination even if the feature as such is not identified fully.

This approach is expected to be useful to decipher the yet unknown contribution of certain known sources to the pattern of PM compounds of a recipient that may result from multiple sources.

### Selection of suspects

In this study, TCR particles are used as source material and a sample of material collected by regular street-cleaning activity (road dust, RD) is used as environmental sample close to the source. Tire material from the tread is abraded from tires while driving on streets; together with road material and particulate material deposited on the road, this mixture is called TRWP [9]. A part of this material resides on the street and ages (sun, temperature, shear forces) before it is removed by road cleaning. Such road dust should contain aged TRWP, together with particulate matter from other sources. Therefore, RD was selected as the “close to source” sample for this exercise.

As this study aims at persistent and mobile chemicals, leachates rather than extracts of the two materials are analyzed. With this approach, it should be possible to detect tire constituents that are persistent and mobile compounds released from tire material after deposition in the aqueous environment.

**Fig. 2:**
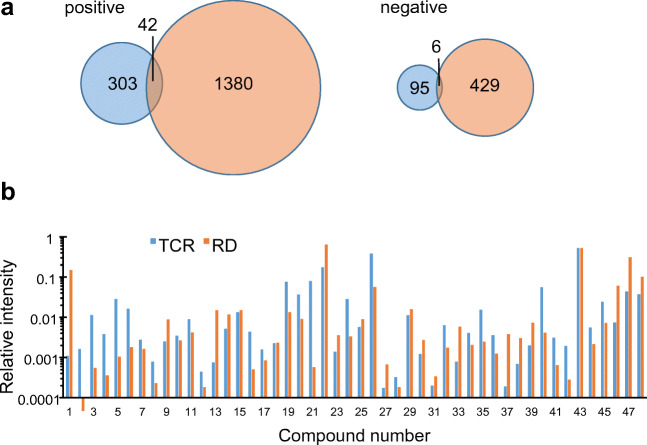
**a** Venn diagrams of the features determined either in the tire crumb rubber (TCR, blue) or in the road dust (RD, orange) and in both samples; left: positive mode, right: negative mode. **b** Signal intensity of the 48 suspects in TCR and RD, normalized to the total intensity of these signals in either sample. no. 1–no. 42 recorded in positive mode, no. 43–no. 48 in negative mode

In the TCR leachate, 303 signals in positive mode and 95 signals in the negative mode were detected by LC-HRMS which were significantly higher than in the blank sample, while 1380 (pos) and 429 (neg) such signals were detected in the RD leachate (Fig. 2a). The generally lower number of features in negative mode corresponds to the higher selectivity of this ionization mode. Both leachates had a total of 42 (pos) and 6 (neg) signals in common, suggesting that these are tire-derived components of environmental relevance. The filter effect of combining a source sample with a “close to source” sample is enormous: in this case, it reduces the number of features of the source (TCR) that merit further attention by 88%.

The number of features detected in each of the sample and shared by both samples may have been higher, if the leachates would have been enriched prior to LC-HRMS analysis. But as this was a conceptual study, enrichment was not performed. Furthermore, enrichment of very polar analytes from water is delicate and may not be complete.

The pattern of relative signal intensities for the 48 suspects in the two samples, normalized to the cumulative signal intensity of all suspects in the respective sample, is quite different (Fig. 2b). For example, the relative intensity of compound nos. 2–6, 21, 40, and 47 is about one order of magnitude higher in TCR than in RD, while nos. 1, 22, and 47 are much more prominent in the latter.

These differences may either reflect different environmental stabilities of the compounds—less stable tire components may be depleted already in the environmental sample close to the source (RD), while the proportion of transformation products may increase from TCR to RD—or it indicates the presence of other urban sources from which a TCR-related compound is introduced into road dust and which increase its proportion in RD compared with TCR.

### Identification of suspects

The detected suspects cover a broad range of molecular masses (*m*/*z* 94–553) and polarity (RT 1.36–9.72 min). Their tentative identification was performed based on their mass spectrometric data (molecular ion plus fragment ions) and verified by standard compounds, if possible. The identified compounds belong to four classes of chemicals (Table 1). For 10 suspects, only the molecular formulas were confirmed (Table 1), while for seven suspects (no. 12, no. 32, no. 37, no. 38, no. 44, no. 47, and no. 48), not even an unambiguous formula could be derived. Still, most of these 17 compounds could be ascribed to one of the contaminant classes (Table 1).

**Table 1:** Tire-related suspects, their classification, and basic identification data (for more details, refer to ESM Table S2)

Suspect no.	Class	*m*/*z* for cation/anion	RT (min)	Proposed cation formula	Proposed compound	Identification confidence*	Reference
1	(methoxymethyl)Melamines	553.2832	6.83	C_19_H_42_N_6_O_11_Na	Unknown	3	
2		473.2323	9.32	C_17_H_34_N_6_O_8_Na	Diformylated HMMM (HMMM+2×CH_2_O)	2	13
3		443.2230	9.01	C_16_H_32_N_6_O_7_Na	Formylated HMMM	2	13, 14
4		429.2057	8.18	C_15_H_30_N_6_O_7_Na	Diformylated PMMM	3	
5		413.2133	8.64	C_15_H_30_N_6_O_6_Na	Hexa(methoxymethyl)melamine (HMMM)	1	13, 14
6		399.1960	7.75	C_14_H_28_N_6_O_6_Na	HMMM TP 377	2	13
7		385.1818	6.95	C_13_H_26_N_6_O_6_Na	Unknown	3	
8		369.1862	7.61	C_13_H_26_N_6_O_5_Na	Penta(methoxymethyl)melamine (PMMM)	2	13, 14
9		349.1601	6.32	C_13_H_22_N_6_O_4_Na	Tetra(methoxymethyl)melamine	2	13, 14
10		311.1435	5.20	C_10_H_20_N_6_O_4_Na	HMMM TP 311	2	13
11		215.1244	3.03	C_7_H_15_N_6_O_2_	Di(methoxymethyl)melamine	2	13
12	Amines	351.1315	5.75	Unknown	Unknown	5	
13		310.1179	4.09	C_17_H_16_N_3_O_3_	Unknown	3	
14		284.1389	6.20	C_16_H_18_N_3_O_2_	Unknown	3	
15		266.1283	4.62	C_16_H_16_N_3_O	Unknown	3	
16		249.1571	4.57	C_12_H_22_N_2_O_2_Na	Cyclic PA6 dimer	3	
17		248.1636	5.80	C_15_H_22_NO_2_	Unknown	4	
18		226.1226	9.58	C_15_H_16_NO	Dimethylamino-benzophenone	4–5	
19		225.1967	9.72	C_13_H_25_N_2_O	1,3-Dicyclohexylurea	3	14
20		219.1486	9.19	C_13_H_19_N_2_O	*N*-Cyclohexyl-*N*′-phenylurea	3	14
21		196.207	5.62	C_13_H_26_N	*N*-Methylcyclohexylamine	3	14
22		182.191	5.85	C_12_H_24_N	Dicyclohexylamine	3	
23		213.1015	7.99	C_13_H_13_N_2_O	Benzamidin (loss of ammonia)	3	27
24		212.1190	4.95	C_13_H_14_N_3_	Diphenylguanidine	1	27, 30
25		211.1243	8.49	C_12_H_16_N_2_Na	Unknown	4	
26		186.2204	6.02	C_12_H_28_N	Tributylamine	4	
27		136.0745	5.81	C_8_H_10_NO	Acetanilide	4	
28		128.1439	2.89	C_8_H_18_N	Cyclohexylethylamine	4	
29		114.0908	4.17	C_6_H_12_NO	Caprolactam	4	
30		94.0641	1.36	C_6_H_8_N	Unknown	4	
31	PEGs	377.2139	6.72	C_16_H_34_O_8_Na	Heptaethylene glycol monoethyl ether	4	
32		395.1578	6.28	C_14_H_30_NO_8_NaS	Unknown	4	
33		333.1880	5.92	C_14_H_30_O_7_Na	Hexaethylene glycol monoethyl ether	4	
34		289.1618	5.47	C_12_H_26_O_6_Na	Hexaoxaoctadecane/propoxylated glycerol	4	
35		289.1618	5.81	C_12_H_26_O_6_Na	Hexaoxaoctadecane/propoxylated glycerol	4	
36		245.1353	4.83	C_10_H_22_O_5_Na	Tetraethylene glycol dimethyl ether	4	14
37	Others	249.0085	6.56	Unknown	Unknown	5	
38		158.1513	8.96	Unknown	Unknown	5	
39	Benzothiazoles	167.9930	7.48	C_7_H_5_NS_2_	Mercaptobenzothiazole	1	17
40		152.0158	7.05	C_7_H_6_NOS	Hydroxybenzothiazole	1	17
41		151.0319	3.93	C_7_H_7_N_2_S	Aminobenzothiazole	1	17
42		136.0203	7.19	C_7_H_6_NS	Benzothiazole	1	17
43		213.9645	4.80	C_7_H_4_NO_3_S_2_	Benzothiazole sulfonic acid	1	20
44	Others	178.0537	4.16	Unknown	Unknown	5	
45		121.0294	4.73	C_7_H_5_O_2_	Benzoic acid	5	
46		173.1188	7.56	C_9_H_17_O_3_	Unknown	5	
47		309.1026	8.71	Unknown	Unknown	5	
48		309.1027	9.48	Unknown	Unknown	5	

*Identification confidence: (1) comparison with standard; (2) RT, exact mass, fragment ions agree with literature [13]; (3) proposed based on elemental composition, supported by fragmentation; (4) proposed based on elemental composition, no fragments; (5) several molecular formulas possible

#### HMMM and related compounds

The identification of hexamethoxymethyl melamine (HMMM, no. 5) and other methoxymethyl melamines (MMMs) is based on the two characteristic fragment ions *m*/*z* 177.0876 (C_7_H_9_N_6_) and *m*/*z* 163.0715 (C_6_H_7_N_6_) and for the smaller MMMs on *m*/*z* 139.0699 (C_4_H_7_N_6_) and *m*/*z* 165.0877 (C_6_H_9_N_6_). Eight of these compounds correspond to those detected and identified previously in a biodegradation experiment of HMMM [14]. Three further compounds of this group have not been reported before, but show the same characteristic fragments (no. 1, no. 4, no. 7). Of these, the molecular formula of no. 7 corresponds to an oxidation product of PMMM, while no. 1 may be an adduct of no. 7, due to the occurrence of no. 7 as one of its fragment ions (see Electronic Supplementary Material (ESM) Table S1).

HMMM and related compounds are contained in melamine-based resins and employed in tire production as curing agents, replacing widely used phenolic resins. HMMM is reported to improve the adhesion of tire rubber to textile and steel cord [17]. HMMM and four related substances were recently determined in road runoff, in tire leachate and in an urban creek around Seattle (USA) [15] and, together with eight TPs, in a dedicated study on HMMM biodegradation and occurrence in an urban water cycle in Germany [14].

These data now prove that the number of HMMM-related compounds possibly originating from TCR is significantly larger. The other MMMs detected in the TCR and RD samples, namely TMMM and DMMM, belong to the earlier TPs of HMMM as recently shown in a biodegradation experiment [14]. This corresponds to the limited exposure of the two materials studied here to environmental microbiota. Even smaller and more polar TPs of this family have recently been shown to be true PM compounds that can pass through bank filtration and reach raw waters used for drinking water production [14].

#### Benzothiazole

The identity of the four benzothiazoles (no. 39–no. 42, Table 1) was confirmed by standards, by agreement in retention time and exact mass of the molecular ions. Due to their low molecular mass, characteristic fragment ions for further confirmation could not be detected by Q-TOF-MS.

Some benzothiazoles, for example, *N*-cyclohexylbenzothiazole-2-sulfenamide (DCBS), dibenzothiazolyldisulfide (MBTS), and 2-4-(morpholinyl-benzothiazole), are used in rubber production as vulcanization accelerators. A series of TPs of these vulcanization accelerators has frequently been reported in the context of tires and road runoff: benzothiazole itself appears to be the first compound proposed as a marker for tire input into sediments [13]. Another three benzothiazoles were found here in positive mode, amino benzothiazole (NH2-BT), hydroxy-benzothiazole (HOBT), and mercaptobenzothiazole (MBT), and benzothiazole sulfonic acid (BTSA) in negative mode.

Not all of these compounds will survive for long in the environment. BT, NH2-BT, and HOBT have been shown to be biodegradable, while MBT may either be oxidized to MBTS or methylated to the more stable and volatile methylthiobenzothiazole [19]. Anyhow, some benzothiazoles have been found widely in effluents of WWTPs [20] and surface waters [21]. Release from rubber, however, is not the only possible source of benzothiazoles found in the aqueous environment; they may also stem from other sources, e.g., from the fungicide thiocyanomethylthiobenzothiazole (TCMTB) [19]. BTSA, BT, HOBT, and MTBT have previously been quantified in road runoff after rainfall events [22, 23]; of these, BTSA proved most prominent and most stable in the environment.

#### Amines

Furthermore, 19 amines were found in the data set (no. 12–no. 30, Table 1). For 9 of these, structure proposals were elaborated and molecular formulas proposed for another 10 compounds. Six of these compounds were also detected in the blank sample, but at significantly lower signal intensity (see ESM Table S2).

The exact mass of the cation of no. 19 (*m*/*z* 255.1697) only fits to the formula [C_13_H_25_N_2_O]+, but Chemspider reports a total of 15,030 different molecules for this molecular ion. The recorded fragment ions *m*/*z* 100.111 ([C_6_H_14_N]^+^, aniline) and *m*/*z* 83.085 ([C_6_H_11_]^+^) do not only support the molecular formula but also point to dicyclohexylurea as molecule, because the same fragments were reported by mzcloud (https://www.mzcloud.org/). Furthermore, dicyclohexylurea was recently reported to be present in tire-related samples and to be known as by-product during surface-grating of polymers [15, 24].

The same fragment ions as for dicyclohexylurea were detected for no. 26, with a formula of [C_12_H_24_N]^+^ (*m*/*z* 182.1910). Both fit well to dicyclohexylamine. Dicyclohexylamine is expected to be formed by hydrolysis from the vulcanization accelerator DCBS. [25]

Aniline (*m*/*z* 100.111; [C_6_H_14_N]^+^) was also detected as fragment ion of no. 16. The second fragment ion ([C_12_H_21_N_2_O]^+^) together with the loss of water points to a formula (Table 1) for which 18,000 possible structures were found in Chemspider. Nevertheless, the tire origin of this compound makes it likely that no. 16 is the cyclic dimer of polyamide (PA6). The PA6 monomer (ε-caprolactam) with *m*/*z* 114 does occur in the suspect list, too. The occurrence of ε-caprolactam and its cyclic dimer is likely because it is used as tire cord [26].

The exact mass and assigned molecular formula of no. 21 were previously reported in highway runoff and proposed to be 3-aminobenzanilide [27]. However, the fragmentation pattern of the isomer 4-aminobenzanilide, which was available as standard, was quite different. While the molecular formula would also fit for diphenylurea, the spectrum of no. 21 did not agree to its data in mzcloud. Based on the neutral losses of ammonia, C_6_H_5_ and C_6_H_7_NO, no. 21 is proposed to be structurally related to *N,N*′-diphenylguanidine (DPG; loss of ammonia and C_6_H_7_N), where the oxygen is directly connected to the aromatic ring. The product ion spectrum of no. 15 suggests that this compound is also related to DPG, with its aliphatic amino group substituted by C_3_H_5_NO. This C_3_H_5_NO unit may originate from the solvent dimethylformamide (C_3_H_7_NO) frequently used in amide production.

The identity of DPG (no. 22) was confirmed by a standard. DPG is used in tire rubber as a secondary accelerator in silicon tread mixes (also called “activator”) [9, 28] and was previously shown to be released from tires [12]. Information on the environmental stability of DPG is limited but contradictory: while DPG was reported not to be stable in terrestrial environment [12] and to be degraded in a production wastewater (though data were not provided) [29], another study found DPG to be stable in a lab degradation experiment [30].

Besides DPG, the bicyclic amines 1,3-dicyclohexylurea (no. 26), *N*-methyl-dicyclohexylamine (no. 24), and *N*-cyclohexyl-*N*′-phenylurea (no. 20) were detected here, in agreement with their recent detection in road runoff [15]. Compound no. 28 is probably cyclohexylethylamine as this has been reported to be found in rubber products like tires by ECHA [31]. Chemspider, however, proposes 1184 alternative structures for this molecular formula. Compound no. 25 is probably tributylamine as it is used in the manufacture of vulcanization accelerators [32] and no. 30 is suggested to be aniline and thus a transformation product of *N*-cyclohexyl-*N*′-phenylurea [15].

#### Glycols

A collection of glycols were also detected and identified (no. 31–no. 36), among them polyethylene glycol, hexaethylene glycol monoethyl ether, and tetraethylene glycol dimethyl ether. All these were identified based on the formulas of their sodium adducts (Table 1), the lack of fragment ions typical for sodium adducts, and the absence of protonated molecular ions. A series of polyglycols were reported earlier in tire leachates [15]. As glycols are widely used chemicals, their diagnostic value is limited.

### Application to suspect screening

#### Screening in an urban creek prior to and after an incident of combined sewer overflow

In urbanized areas with combined sewer systems, the runoff from roads and other impervious areas is collected together with household wastewater and other wastewaters in the sewer system and treated in municipal wastewater treatment plants. This minimizes the effect of road runoff on the environment. In case of strong rainfall, however, the hydraulic capacity of the sewer system and the WWTP can be exceeded and untreated wastewater may have to be discharged directly into urban rivers and creeks, without proper treatment (combined sewer overflow, CSO) [33].

While the major concern with such CSOs may be discharge of pathogens to and oxygen depletion in the receiving water, also the concentration of traffic-related chemicals can be expected to increase after such an incident. As a proof of concept, the suspect list derived from the above exercise was used to search for tire-related trace contaminants in the LC-HRMS data of an urban creek before and after a strong rainfall accompanied by a CSO.

Indeed, 41 of the 48 suspects were detected in the urban creek subsequent to the CSO, while 27 suspects were detected already at dry weather (see ESM Table S2). Obviously, the urban creek is affected by tire-related trace contaminants and this influence is strengthened by a CSO. The same creek has previously been shown to exhibit increasing microplastic loads after rain events [16].

The high percentage of positive findings (85% and 56%) in a limited number of samples (3) is very unusual for a suspect screening exercise. This is a clear benefit of the source-related smart suspect screening that effectively directs the attention to the compounds of interest.

Depending on the extent to which the concentration of the CSO-related compounds increased in the creek, these 41 compounds were ascribed to two groups: group 1 includes all suspects for which the concentration after the CSO increased by more than one order of magnitude. Prominent members of this group are HMMM and most of the related substances such as PMMM, TMMM, and DMMM, three cyclohexyl amines, and the glycols (Table 2).

**Table 2:**
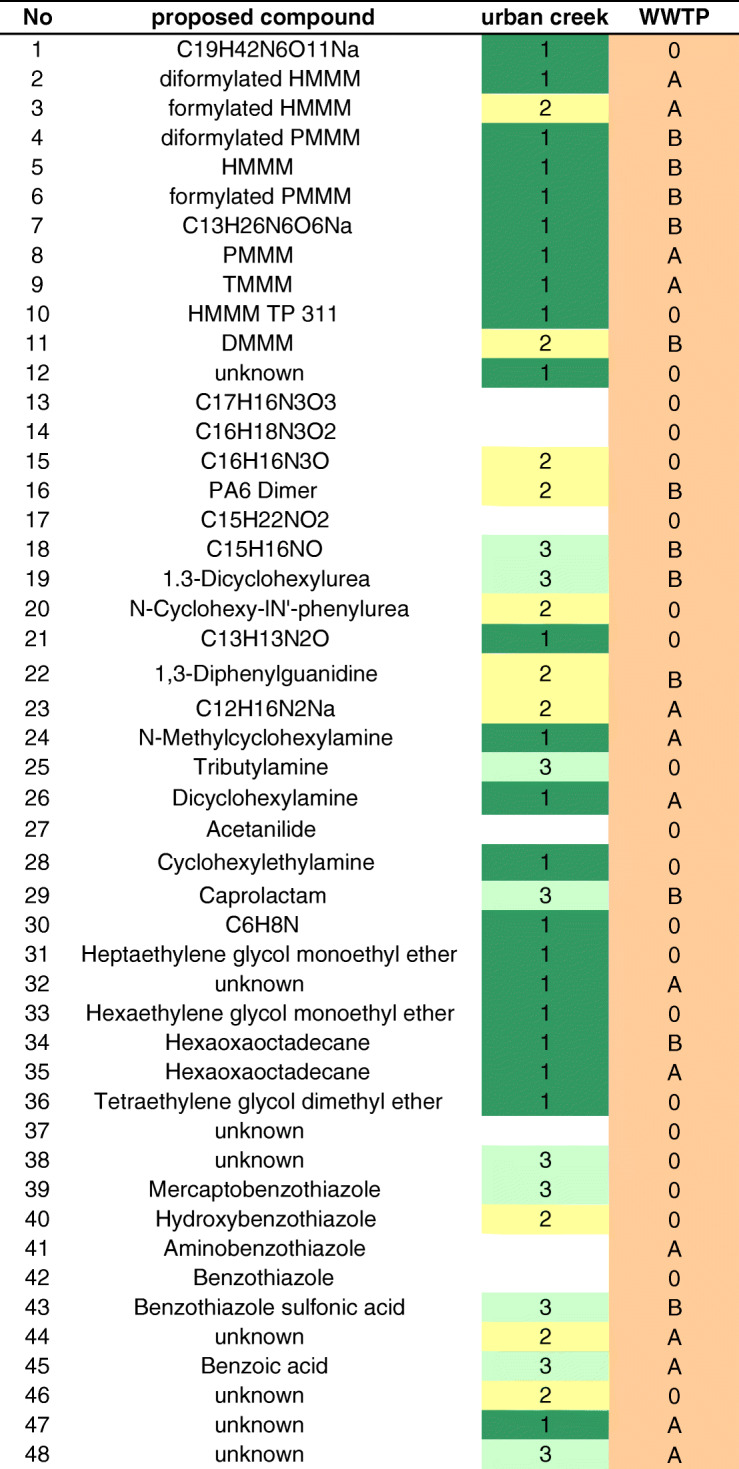
Tire-related suspects detected in an urban creek and a WWTP. Numbers (and colors) denote groups of compounds with similar concentration profile; creek 1, increase > factor 10 after CSO; 2, increase < factor 10 after CSO; 3, no increase; WWTP, A, median removal > 70%; B, median removal < 70% (*n* = 3)

Group 2 combines all tire-related chemicals with an increase by less than one order of magnitude. The urea compounds and DPG, as well as one of the benzothiazoles, namely HOBT, belong to this group (Table 2). Group 3 compounds did not show a signal increase. But even for these compounds, the load in the creek increased by the CSO as the water flow increased by more than a factor of 3 [16]. BTSA is a member of this group.

There may be various reasons why not all tire-derived compounds show the same CSO-dependent increase in the receiving urban creek. Less persistent compounds may increase more strongly, when untreated CSO is directly discharged into surface water as these compounds are usually removed in WWTPs before discharge. But other sources than tire wear and road runoff may further modulate this intensity pattern.

#### Screening WWTP influent and effluent

During a CSO event, untreated municipal wastewater reaches surface water and discharges unstable compounds which are usually eliminated in WWTP under regular operational conditions. During less heavy rainfall, the surface runoff is directed to WWTPs. Therefore, one can expect contaminants originating from TRWP to also show up in the influent of WWTPs.

This was tested by applying the same suspect list to LC-HRMS data of three influent samples of a WWTP [14]. Here, 32 of the 48 suspects (67%) were detected in at least one of three influent samples (see ESM Table S2). Again, this is a remarkably high frequency of detection. However, LC-HRMS screening approaches do not offer highest sensitivity for all suspects. This, likely, is the explanation why benzothiazoles were detected in only a few of the samples. With a more sensitive approach, the percentage of positive detections would have been even higher, likely.

Those compounds with highest signal intensity in the WWTP influent (HMMM, dicyclohexylamine; see ESM Table S2) were also very prominent in the TCR sample. DPG, however, a compound with high contribution in TCR and in RD, was not strong in the WWTP influent.

With the source-related smart suspect screening, a significant number of trace organic contaminants that are released from tires as well as from road dust were found in municipal wastewater. However, these compounds may also have other sources than TRWP. A monitoring of road runoff, household wastewater, and industrial discharges would allow to assess the quantitative relevancy of other sources compared with TRWP.

A comparison of the signal intensities of the compounds in the samples collected from influent and effluent of the WWTP provides an indication for the extent of elimination of these tire-related compounds. Based on their signal intensity, 10 of these compounds exhibited a median elimination of more than 70% (group A), while another 12 showed elimination of less than 70% (group B; Table 2).

Differences in the matrix effects for one analyte in influent and effluent samples are likely to occur and, therefore, a comparison of signal intensities can be biased. However, these data suggest that TRWP can release organic contaminants that are sufficiently persistent and mobile to escape from WWTPs.

## Conclusion

The concept of source-related smart suspect screening by LC-HRMS consists of two major steps.

First, a source sample and a “close to source” sample are analyzed in a non-targeted manner and the data used to generate a suspect list. For the study of tire-related organic compounds in environmental samples, TCR and RD leachates were used in this step. For this set of samples, this process turned out to be quite efficient: the effort of elaborating structure proposals and of structure verification could be focused to a number of 42 compounds clearly related to TRWP. And the knowledge that the suspects originated from tires supported the elaboration of structure proposals.

Second, the relatively short suspect list is applied to environmental samples, in this case an urban creek affected by CSO and municipal wastewater before and after treatment. A very high percentage of positive findings occurred, up to 85% for the urban creek and 67% for the municipal wastewater. This illustrates that the first step of the approach resulted in a list of polar suspects that was of high relevance for the aqueous environment.

The search for persistent and mobile substances originating from tires by source-related smart suspect screening outlines that a complex pattern of such organic contaminants may be introduced into surface waters by discharges of treated municipal wastewater of combined sewer systems as well as by combined sewer overflows.

Source-related smart suspect screening appears to be a useful approach in search of the origin of persistent and mobile substances that are introduced into the water cycle.

## Electronic supplementary material

ESM 1 (PDF 147 kb)
