# Supplementary material for: Source-related smart suspect screening in the aqueous environment: search for tire-derived persistent and mobile trace organic contaminants in surface waters
Source: Anal Bioanal Chem. 2020 May 8;412(20):4909–19. doi: 10.1007/s00216-020-02653-1 (PMC7334239; doi:10.1007/s00216-020-02653-1)
Supplement: Supplementary file 1 — (PDF 147 kb) [file 216_2020_2653_MOESM1_ESM.pdf]

Analytical and Bioanalytical Chemistry

Electronic Supplementary Material

Source-related smart suspect screening in the aqueous environment: search for tire-derived persistent and mobile trace organic contaminants in surface waters

Bettina Seiwert, Philipp Klöckner, Stephan Wagner, Thorsten Reemtsma

Table S1 Compounds Identified in TCR and RD samples with fragment ions and the assigned elemental composition

number	proposed compound	*m/z* of molecular ion or adduct ion	RT [min]	*m/z* of fragment ions	assigned elemental composition
**Positive mode**		$[M+H]^+$ / $[M+Na]^+$		cations	
1	C19H42N6O11Na	553.283	6.83		C19H42N6O11Na
				385.1818	C13H26N6O6Na
				337.2134	C12H22N6O4Na
				283.1531	C11H19N6O3
				177.0876	C7H9N6
				163.0715	C6H7N6
2	diformylated HMMM	473.232	9.32		C17H34N6O8Na
				207.1003	C8H11N6O
				177.0876	C7H9N6
				163.0715	C6H7N6
3	formylated HMMM	443.223	9.01		C16H32N6O7Na
				315.1776	C12H23N6O4
				283.152	C11H19N6O3

				239.126	C9H15N6O2
				177.0890	C7H9N6
4	diformylated PMMM	429.206	8.18		C15H30N6O7Na
				399.1965	C14H28N6O6Na
				367.1700	C13H24N6O5Na
				283.1521	C11H19N6O3
				239.1243	C9H15N6O2
				207.0983	C8H11N6O
				177.0876	C7H9N6
				163.0715	C6H7N6
5	HMMM	413.213	8.64		C15H30N6O6Na
				359.2056	C14H27N6O5
				315.1788	C12H23N6O4
				283.1520	C11H19N6O3
				239.1263	C9H15N6O2
				207.1003	C8H11N6O
				177.0889	C7H9N6

				163.0735	C6H7N6
6	formylated PMMM	399.196	7.75		C14H28N6O6Na
				369.1862	C13H26N6O5Na
				337.1593	C12H22N6O4Na
				315.1784	C12H23N6O4
				283.1511	C11H19N6O3
				239.1243	C9H15N6O2
				207.0983	C8H11N6O
				177.0876	C7H9N6
				163.0715	C6H7N6
7	C13H26N6O6Na	385.182	6.95		C13H26N6O6Na
				283.1531	C11H19N6O3
				177.0876	C7H9N6
				163.0715	C6H7N6
8	PMMM	369.186	7.61		C13H26N6O5Na
				315.1784	C12H23N6O4
				239.1263	C9H15N6O2

				177.0876	C7H9N6
				163.0715	C6H7N6
9	TMMM	349.154	6.32		C13H22N6O4Na
				325.1592	C11H22N6O4Na
				257.1339	C9H17N6O3
				227.124	C8H15N6O2
				195.0979	C7H11N6O
				165.0875	C6H9N6
				151.0715	C5H7N6
10	HMMM TP 311	311.144	5.20		C10H20N6O4Na
				165.0877	C6H9N6
				151.0727	C5H7N6
11	DMMM	215.124	3.03		C7H15N6O2
				201.1087	C6H13N6O2
				151.0727	C5H7N6
				139.0699	C4H7N6
12	unknown	351.132	5.75		unknown

				305.1274<br>235.0798<br>193.0695	-CH2O2<br>-C6H8N<br>-C6H8N.-C2H2O
13	C17H16N3O3	310.118	4.09	 250.0984	C17H16N3O3<br>C15H12N3O
14	C16H18N3O2	284.139	6.20	 145.075<br>195.091	C16H18N3O2<br>C9H9N2<br>C13H11N2
15	C16H16N3O	266.128	4.62	 195.091<br>92.049	C16H16N3O<br>C13H11N2<br>C6H6N
16	PA6 Dimer*	249.157	4.57	 209.165<br>100.1110	C12H22N2O2Na<br>C12H21N2O<br>C6H14N
17	C15H22NO2	248.164	5.80		
18	C15H16NO	226.123	9.58		
19	1.3-dicyclohexylurea	225.197	9.72		C13H25N2O

				100.111<br>83.085	C6H14N<br>C6H11
20	N-Cyclohexyl-N'-phenylurea	219.149	9.19	 137.0699<br>120.0443<br>94.0641	C13H19N2O<br>C7H9N2O<br>C7H6NO<br>C6H8N
21	C13H13N2O	213.102	7.99	 184.0760<br>108.068	C13H13N2O<br>C12H10NO<br>C6H8N2
22	1.3-Diphenylguanidine	212.119	4.95	 195.0917<br>94.0641	C13H14N3<br>C13H11N2<br>C6H8N
23	C12H16N2Na	211.124	8.49		
24	N-Methylcyclohexylamine	196.207	5.62	 114.127<br>83.085	C13H26N<br>C7H16N<br>C6H11
25	Tributylamine	186.220	6.02		

26	Dicyclohexylamine	182.191	5.85	100.111<br>83.086	C12H24N<br>C6H14N<br>C6H11
27	C8H10NO_Acetanilide ?	136.075	5.81		
28	cyclohexylethylamine	128.144	2.89		
29	caprolactam	114.091	4.17		
30	C6H8N	94.064	1.36		
31	heptaethylene glycol monoethyl ether	395.158	6.28	333.1880<br>175.0795	C14H30NO8NaS<br>-NOS<br>-C6H16O5
32	unknown	377.214	6.72		
33	hexaethylene glycol monoethyl ether	333.188	5.92		
34	hexaoxaoctadecane/ propoxylated glycerol	289.162	5.47		
35	hexaoxaoctadecane/ propoxylated glycerol	289.162	5.81		
36	tetraethylene glycol dimethyl ether	245.135	4.83		
37	unknown	249.009	6.56		

38	unknown	158.151	8.96		
39	mercaptobenzothiazole	167.993	7.48		
40	hydroxybenzothiazole	152.016	7.05		
41	aminobenzothiazole	151.032	3.93		
42	benzothiazole	136.020	7.19		
negative mode		$[M-H]^-$		anions	
43	benzothiazolsulfonic acid	213.9645	4.80	121.0293	C7H4NS
44	unknown	178.0537	4.16		
45	C7H5O2	121.0294	4.73		
46	C9H17O3	173.1188	7.56		
47	unknown	309.1026	8.71		
48	unknown	309.1027	9.48		

Table S2 Detected relative peak areas in the TCR and the RD samples, in River Parthe at dry weather, after some and after heavy rainfall, and in the influent and effluent of a wastewater treatment plant (3 dates)

Compound No	TCR	RD	River Parthe			Wastewater treatment plant					
			Dry weather	some rain	strong rain	influent_ 1	influent_ 2	influent_ 3	effluent_ 1	effluent_ 2	effluent_ 3
1	948	72365	n.d.	1002	3736	n.d.	n.d.	n.d.	n.d.	n.d.	n.d.
2	1414	23	n.d.	n.d.	17372	56	113	1796	111	11	292
3	9899	268	28005	32224	96285	2596	3075	16906	2552	385	5223
4	3273	173	n.d.	n.d.	26686	280	908	833	110	28	392
5	24858	510	2548793	5213327	170204333	18823	18775	50038	10446	2050	17057
6	14205	883	243	250	51177	4336	8645	6407	1255	341	3063
7	2385	792	n.d.	n.d.	9874	496	1596	277	99	45	199
8	680	112	n.d.	n.d.	42863	3136	1385	10420	1463	61	3278
9	2171	4297	n.d.	n.d.	7432	1450	2050	5722	1085	168	1036
10	3029	1289	n.d.	n.d.	3738	32	49	65	n.d.	13	n.d.
11	7764	2044	2124	2133	17458	553	2908	1044	1135	212	1693

**12**	**388**	**89**	**n.d.**	**n.d.**	**11541**	**n.d.**	**n.d.**	**n.d.**	**n.d.**	**n.d.**	**n.d.**
**13**	**651**	**7296**	**n.d.**	**n.d.**	**n.d.**	**n.d.**	**n.d.**	**n.d.**	**n.d.**	**n.d.**	**n.d.**
**14**	**4466**	**5706**	**n.d.**	**n.d.**	**n.d.**	**n.d.**	**n.d.**	**n.d.**	**n.d.**	**n.d.**	**n.d.**
**15**	**11503**	**7328**	**343**	**1216**	**1616**	**n.d.**	**7**	**n.d.**	**n.d.**	**14**	**n.d.**
**16**	**3810**	**247**	**923900**	**1185208**	**2417123**	**864**	**674**	**957**	**373**	**375**	**347**
**17**	**1367**	**412**	**n.d.**	**n.d.**	**n.d.**	**n.d.**	**n.d.**	**n.d.**	**n.d.**	**n.d.**	**n.d.**
**18**	**1971**	**1133**	**1798**	**1607**	**1843**	**29**	**32**	**21**	**33**	**29**	**23**
**19**	**65912**	**6537**	**12922**	**13080**	**12122**	**4279**	**4503**	**3662**	**4580**	**4501**	**4717**
**20**	**31689**	**4422**	**306**	**686**	**764**	**n.d.**	**16**	**n.d.**	**n.d.**	**14**	**n.d.**
**21**	**68854**	**281**	**n.d.**	**n.d.**	**1015**	**n.d.**	**n.d.**	**n.d.**	**n.d.**	**n.d.**	**n.d.**
**22**	**149261**	**311422**	**15035**	**15815**	**25462**	**438**	**2201**	**250**	**179**	**703**	**144**
**23**	**1202**	**1747**	**1924**	**7272**	**13434**	**2949**	**65**	**523**	**30**	**38**	**24**
**24**	**24833**	**1626**	**522**	**397**	**32299**	**190**	**10**	**4256**	**14**	**n.d.**	**318**
**25**	**4982**	**4386**	**738**	**763**	**812**	**6**	**9**	**10**	**n.d.**	**n.d.**	**n.d.**
**26**	**333997**	**27566**	**2565**	**2707**	**402762**	**64196**	**4647**	**33321**	**492**	**19**	**455**
**27**	**153**	**330**	**n.d.**	**n.d.**	**n.d.**	**n.d.**	**n.d.**	**n.d.**	**n.d.**	**n.d.**	**n.d.**
**28**	**280**	**89**	**n.d.**	**39591**	**428752**	**n.d.**	**n.d.**	**n.d.**	**n.d.**	**n.d.**	**n.d.**

**29**	**9725**	**7771**	**36218**	**28805**	**34657**	**62141**	**8373**	**35342**	**16220**	**36644**	**22521**
**30**	**1073**	**1339**	**n.d.**	**n.d.**	**1428**	**3575**	**2789**	**3854**	**n.d.**	**n.d.**	**n.d.**
**31**	**174**	**166**	**n.d.**	**n.d.**	**11047**	**n.d.**	**n.d.**	**n.d.**	**n.d.**	**n.d.**	**n.d.**
**32**	**5515**	**851**	**763**	**1827**	**96645**	**752**	**995**	**800**	**n.d.**	**n.d.**	**n.d.**
**33**	**686**	**2833**	**n.d.**	**n.d.**	**10631**	**677**	**67**	**18**	**n.d.**	**n.d.**	**n.d.**
**34**	**3549**	**1008**	**663**	**3714**	**78784**	**75**	**82**	**30**	**13**	**15**	**22**
**35**	**13340**	**1204**	**690**	**1005**	**22963**	**481**	**485**	**180**	**13**	**5**	**8**
**36**	**3088**	**608**	**638**	**700**	**21900**	**751**	**27**	**36**	**n.d.**	**n.d.**	**n.d.**
**37**	**166**	**1840**	**n.d.**	**n.d.**	**n.d.**	**n.d.**	**n.d.**	**n.d.**	**n.d.**	**n.d.**	**n.d.**
**38**	**602**	**1471**	**3180**	**3253**	**2922**	**n.d.**	**n.d.**	**n.d.**	**n.d.**	**n.d.**	**n.d.**
**39**	**1733**	**3537**	**1554**	**1207**	**1278**	**n.d.**	**n.d.**	**n.d.**	**n.d.**	**n.d.**	**n.d.**
**40**	**48672**	**2009**	**523**	**815**	**1406**	**n.d.**	**n.d.**	**n.d.**	**n.d.**	**n.d.**	**n.d.**
**41**	**2710**	**314**	**n.d.**	**n.d.**	**n.d.**	**162**	**22**	**47**	**n.d.**	**n.d.**	**n.d.**
**42**	**1687**	**136**	**n.d.**	**n.d.**	**n.d.**	**n.d.**	**n.d.**	**n.d.**	**n.d.**	**n.d.**	**n.d.**
**43**	**2483**	**60461**	**10031**	**11023**	**9454**	**103**	**125**	**100**	**155**	**179**	**116**
**44**	**27**	**245**	**1520**	**1847**	**5303**	**30616**	**14632**	**28449**	**n.d.**	**n.d.**	**n.d.**
**45**	**115**	**826**	**852**	**936**	**902**	**204**	**n.d.**	**193**	**n.d.**	**n.d.**	**n.d.**

**46**	**35**	**6952**	**187**	**423**	**408**	**n.d.**	**n.d.**	**n.d.**	**n.d.**	**n.d.**	**n.d.**
**47**	**208**	**35395**	**n.d.**	**n.d.**	**29410**	**1171**	**388**	**2036**	**n.d.**	**n.d.**	**n.d.**
**48**	**177**	**11772**	**964**	**1045**	**1180**	**21**	**14**	**39**	**n.d.**	**n.d.**	**n.d.**
